# Perception of Purposeful and Recreational Smartphone Use in Physiotherapy: Randomized Controlled Trial

**DOI:** 10.2196/25717

**Published:** 2021-04-21

**Authors:** Martina Bientzle, Anne Restle, Joachim Kimmerle

**Affiliations:** 1 Leibniz-Institut für Wissensmedien Tuebingen Germany; 2 PT Akademie Tuebingen Tuebingen Germany

**Keywords:** smartphone use, phubbing, physiotherapy, smartphone, therapy, patients, therapists, therapeutic, treatment

## Abstract

**Background:**

Many people constantly use their smartphones in all kinds of situations. Often smartphones are used in a meaningful and targeted way, but frequently they are used as a pastime without any purpose. This also applies to patients and therapists in treatment situations.

**Objective:**

The aim of this study was to investigate how purposeful smartphone use compared with recreational smartphone use (by a physiotherapist or by a patient) influenced the perception of a physiotherapeutic treatment situation. We examined the impact of smartphone use during a physiotherapy session on the perception of the physiotherapist, evaluation of attentiveness, and evaluation of smartphone use in physiotherapy in general.

**Methods:**

Members of various music and sports clubs were invited to participate in an online randomized controlled trial. Participants were randomly assigned to one of four conditions. They watched a video in which a physiotherapeutic treatment was shown and in which a smartphone was used or not used in the following four different ways: (1) therapeutically purposeful use, (2) recreational use by the physiotherapist (looking at the phone from time to time with no therapeutic purpose), (3) recreational use by the patient, and (4) no smartphone use (control condition). After watching the video, the participants indicated their perception of the physiotherapist’s professional competence, social competence, and empathetic behavior. They also rated the physiotherapist’s and patient’s attentiveness and evaluated the usage of smartphones generally in physiotherapy.

**Results:**

The analysis included 118 participants (63 women and 55 men). When the physiotherapist used the smartphone in a purposeful way, the physiotherapist was perceived as more professionally competent (*P*=.007), socially competent (*P*=.03), and empathetic (*P*=.04) than if the physiotherapist used it with no therapeutic purpose. These effects occurred because recreational smartphone use by the physiotherapist was evaluated more negatively than the behavior in the control condition (professional competence: *P*=.001; social competence: *P*=.03; empathy: *P*=.04). Moreover, when the physiotherapist used the smartphone in a recreational way, the physiotherapist was perceived as being less attentive (*P*<.001). Likewise, when the patient used the smartphone in a recreational way, the patient was perceived as being less attentive (*P*<.001). Finally, smartphone use in physiotherapy was rated as more positive in general when the smartphone was used in a purposeful way compared with the conditions in which the physiotherapist or patient looked at the smartphone with no therapeutic purpose (*P*<.001). This positive evaluation occurred because purposeful use led to a more positive rating than no smartphone use (*P*<.001, *R*=0.42).

**Conclusions:**

Smartphones are only appropriate for therapists and patients if they are used directly for a therapeutic purpose. Otherwise, it is better not to use smartphones during treatment.

**Trial Registration:**

AsPredicted (aspredicted.org) #24740; https://aspredicted.org/blind.php?x=vv532i

## Introduction

### Background

Smartphones and other mobile devices are ubiquitous. They enable their users to complete a large number of tasks with little effort. They can be used not only for all kinds of everyday tasks, but also expressly for medical diagnostics [1-4] and therapeutic purposes [5-7]. Whether the use of smartphones is perceived as meaningful and helpful, or rather as unnecessary and even disruptive, certainly depends on the concrete way in which they are used. On the one hand, smartphones are versatile tools that can be used for a variety of tasks and activities like social interactions [8,9], education [10-12], and work-related activities [13,14]. On the other hand, many people are often distracted by their smartphones as they constantly look at them and check whether there is something new, even if this has nothing to do with the actual situation they are in at that moment [15,16]. Despite the positive possibilities offered by smartphones, the devices have dangers. People tend to spend several hours a day with their devices and social interactions can be disturbed [15]. Communication via the smartphone has become faster, easier, and a matter of course. The expectation of being “permanently online and permanently connected” seems to be established in society, in private life, and at work [17-19]. This is particularly noticeable in certain behaviors, such as the frequent habit of briefly “checking” the phone [20-22]. People focusing on their smartphones instead of their physically present communication partners is such a common situation that the term “phubbing” has been created to describe this phenomenon [23-25].

Checking behavior refers to people’s habit of constantly inspecting their smartphones. This includes receiving messages from other people and reports of news through websites or social media. Habits are repetitive procedures and activities in certain situations. This checking habit is a kind of ritual for many people, which mostly happens unconsciously [25,26]. The short repetitive checks are thus automatic behaviors and can be increased by external stimuli, such as visual and auditory stimuli [26]. Constant distraction and interruption can lead to errors and a reduction in efficiency [27]. Negative effects may occur owing to minor interruptions in everyday activities, such as working, learning, and driving. In some cases, smartphone use can even be considered an addiction [28-30]. In everyday life, smartphones accompany many patients and therapists in physiotherapy sessions. As in other situations, smartphones can offer opportunities for therapy [31-33], but at the same time, they can present risks.

### Smartphone Use in Physiotherapy

In physiotherapy, new exercises are often shown to build up muscles or train balance and body awareness. One technique that is often used to support such learning processes is learning through observing one’s own behavior or the behaviors of human role models [34-37]. This includes learning by observing a demonstration of a target movement. Video exercise instruction [38-40], video feedback [41], and video self-modeling [42,43] are possible implementations of this principle.

Through video self-modeling (ie, a video recording of the patient herself/himself during the implementation of a motor movement sequence), visual evidence is produced. This differs from the video exercise instruction, which shows a film excerpt with the motion sequence being performed by an expert or professional. Many studies have confirmed the usefulness of video feedback to improve athletic performance [44,45]. In physiotherapy, a video recording can be used to alert the patient to errors and to request correction of these errors. With the help of smartphones, video recording is made easier and is possible for any patient. Therefore, the video feedback approach with modern smartphones in physiotherapy should be considered as a possible support method.

The interaction between therapists and patients is a complex construct. The therapeutic relationship between therapists and patients is an important factor for the success of physiotherapy. In this type of relationship, aspects, such as social and professional competence and empathy, are particularly important [46-48]. To support successful treatment, attention and motivation are relevant. Distractions of any kind, for example, by a smartphone, can have a negative effect on the success of therapy. In addition, the perceived use of smartphones can influence the therapy. Professional and social competence as well as empathy and attentiveness are factors that contribute to the success of physiotherapeutic treatments. At the same time, these are variables that are potentially affected by the use of smartphones, and accordingly, represent relevant outcome variables for this study [49,50]. A recent study showed that observing the use of smartphones in social interactions influences the perception of smartphone users’ warmth and competence [51]. So far, it is unclear whether this also applies to physiotherapeutic treatment situations and whether the way smartphones are used (purposeful smartphone use vs smartphone use in the sense of a checking habit) influences the perception of the treatment situation and the therapist.

### Outcome Variables and Hypotheses

In order to ensure a positive effect of the therapeutic relationship, *perceived professional competence* of the physiotherapist is central [46,52-54]. The use of a smartphone may influence the perception of the physiotherapist’s professional competence. Targeted and purposeful smartphone use can have a positive effect on the perception of professional competence in cases where use of a smartphone obviously supports therapeutic treatment. On the other hand, smartphone use in the sense of a checking habit can have a negative effect on perceived professional competence, as the device distracts from the treatment and disturbs its progression. On the basis of these considerations, we state the following hypothesis (hypothesis 1): The way the smartphone is used has an impact on participants’ perceived professional competence of physiotherapists. Physiotherapists’ professional competence will be perceived as more pronounced if they use the smartphone in a purposeful way than if they look at it from time to time with no therapeutic purpose.

In addition to professional competence, *perceived social competence* of the physiotherapist is another relevant factor influencing the success of treatment [46,54]. Social competence refers to physiotherapists’ interpersonal and communicative skills. This aspect is important for interpersonal communication and relationship building. A change in the perception of physiotherapists’ social competence with regard to the way they use smartphones is to be expected. Purposeful use of smartphones can have a positive effect on the perception of social competence, when the smartphone serves as an aid during treatment. Conversely, smartphone use in the sense of a checking habit can have a negative effect on perceived social competence. Based on these considerations, we state the following hypothesis (hypothesis 2): The way the smartphone is used has an impact on participants’ perceived social competence of physiotherapists. Physiotherapists’ social competence will be perceived as more pronounced if they use the smartphone in a purposeful way than if they look at it from time to time with no therapeutic purpose.

Adequate empathetic behavior by therapists is also important to promote a positive therapeutic relationship and treatment success. Through *perceived empathetic behavior*, patients feel understood and build a positive relationship and trust with the treating therapist [54-57]. With respect to perceived empathetic behavior, we state the following hypothesis (hypothesis 3): The way the smartphone is used has an impact on participants’ perceived empathetic behavior of physiotherapists. Physiotherapists’ empathetic behavior will be perceived as more pronounced if they use the smartphone in a purposeful way than if they look at it from time to time with no therapeutic purpose.

As an open research question, we examined whether the assumed differences in perceived professional competence, perceived social competence, and perceived empathetic behavior occur owing to the fact that (1) purposeful use of the smartphone leads to a *higher* rating of perceived professional competence, perceived social competence, and perceived empathetic behavior in comparison with no smartphone use or (2) the checking behavior of the physiotherapist leads to a *lower* rating in comparison with no smartphone use.

In order to be able to ensure successful therapy, a good working atmosphere must be created. One factor that can positively influence the working atmosphere is attention. It is important not to be distracted in order to be able to focus one’s full attention on something [58]. Using a smartphone to answer messages, etc, at the same time that an exercise in physiotherapy is being explained can lead to attention problems and consequently to performance degradation [59]. The reason for this is that the attention capacity of a person is not sufficient to perform both tasks at the same time [60,61]. Smartphone use immediately directs attention to the stimuli of the smartphone, disrupting the attention that is needed for the physiotherapy exercise. Thus, we expected the way the smartphone is used to have an impact on how attentively physiotherapists and patients are perceived by the participants. Thus, we state the following hypotheses: hypothesis 4a, physiotherapists will be perceived as being less attentive when they look at the smartphone from time to time with no therapeutic purpose; and hypothesis 4b, patients will be perceived as being less attentive when they look at the smartphone from time to time with no therapeutic purpose.

In this context, it is also relevant to investigate how the concrete use of smartphones in the physiotherapeutic treatment situation affects how the participants evaluate the use of smartphones in general. We state the following hypothesis (hypothesis 5): The way the smartphone is used has an impact on participants’ evaluation of smartphone use in physiotherapy in general. Smartphone use in physiotherapy will be rated as more positive if the smartphone is used in a purposeful way than if physiotherapists or patients look at it from time to time with no therapeutic purpose.

As an open research question, we examined whether the assumed differences are owing to the fact that (1) purposeful use of the smartphone leads to a *more* positive rating in comparison with no smartphone use or (2) use of the smartphone with no therapeutic purpose leads to a *less* positive rating in comparison with no smartphone use.

## Methods

### Ethical Approval

This study was approved by the Ethics Committee of the Leibniz-Institut fuer Wissensmedien (Tuebingen, Germany; approval number: LEK 2019/025).

### Study Design

**Figure 1:**
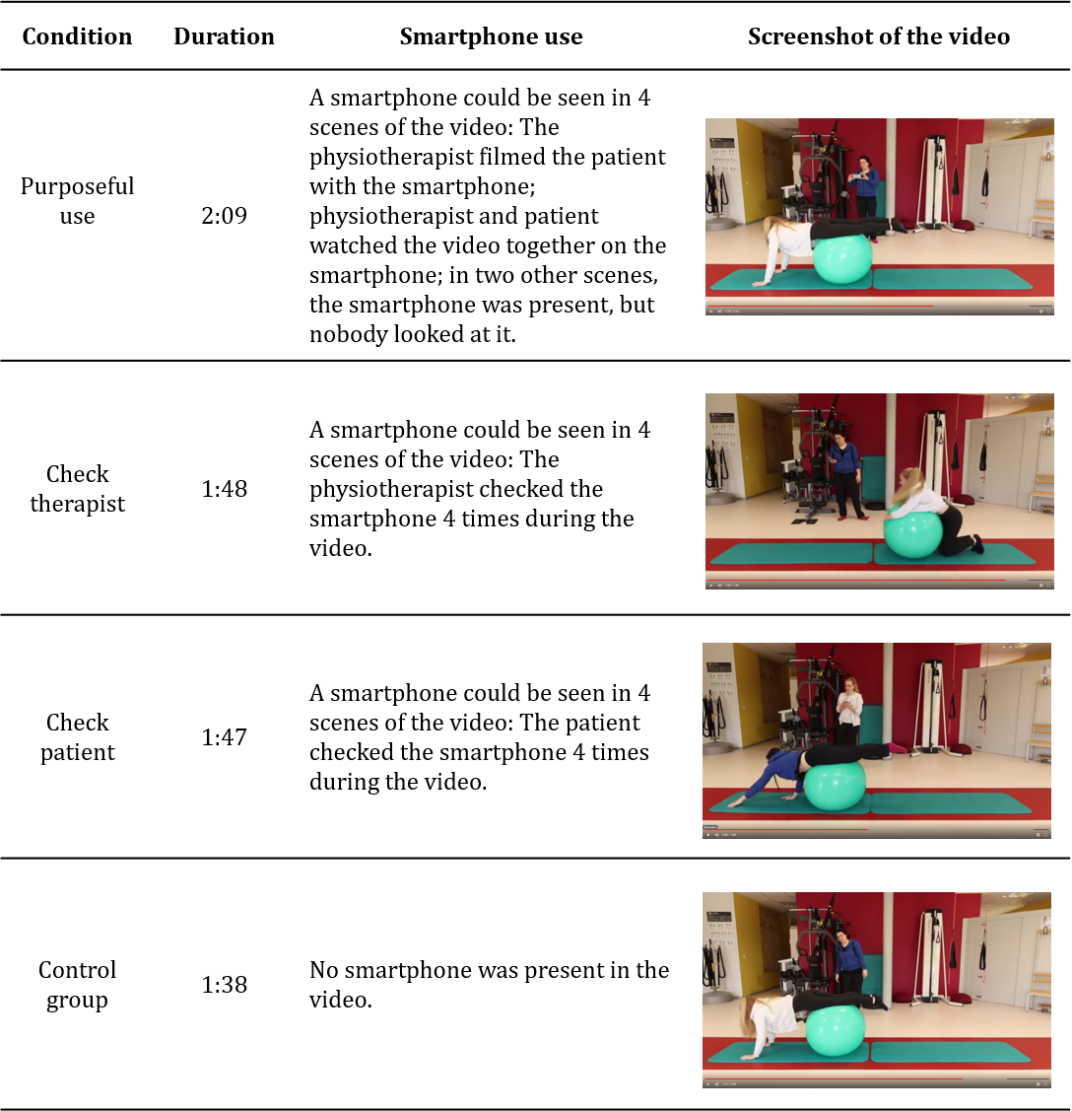
Experimental conditions.

The data for this study were collected in an online survey. The survey contained several questionnaires and a video that differed depending on the experimental condition. We used a video presentation, because this allowed for standardized manipulation of the conditions. The video clips lasted about 2 minutes. They showed a physiotherapeutic treatment with different types of smartphone use, involving a female physiotherapist and a female patient, who were both portrayed by actresses. The first and the last scenes of the video were identical in all of the conditions. First, the patient was greeted and asked about the current back pain. In the following part, the physiotherapist provided instructions for an exercise with a Pezzi ball. Here, the physiotherapist demonstrated the exercise and the patient imitated it. In this part, the conditions differed only with regard to smartphone use (Figure 1). The conditions were as follows: (1) *Purposeful use*, the smartphone was used to support the physiotherapy, in the sense that the patient was filmed with a smartphone doing an exercise to provide qualified feedback; (2) *Check therapist*, the physiotherapist looked at the smartphone from time to time with no therapeutic purpose; (3) *Check patient*, the patient looked at the smartphone from time to time with no therapeutic purpose; (4) *Control*, no smartphone was used during the exercise. At the end of the video, the patient was asked to do the exercise again. This scene was identical in all conditions. The participants were randomly assigned to watch one of the four videos.

### Participants

**Figure 2:**
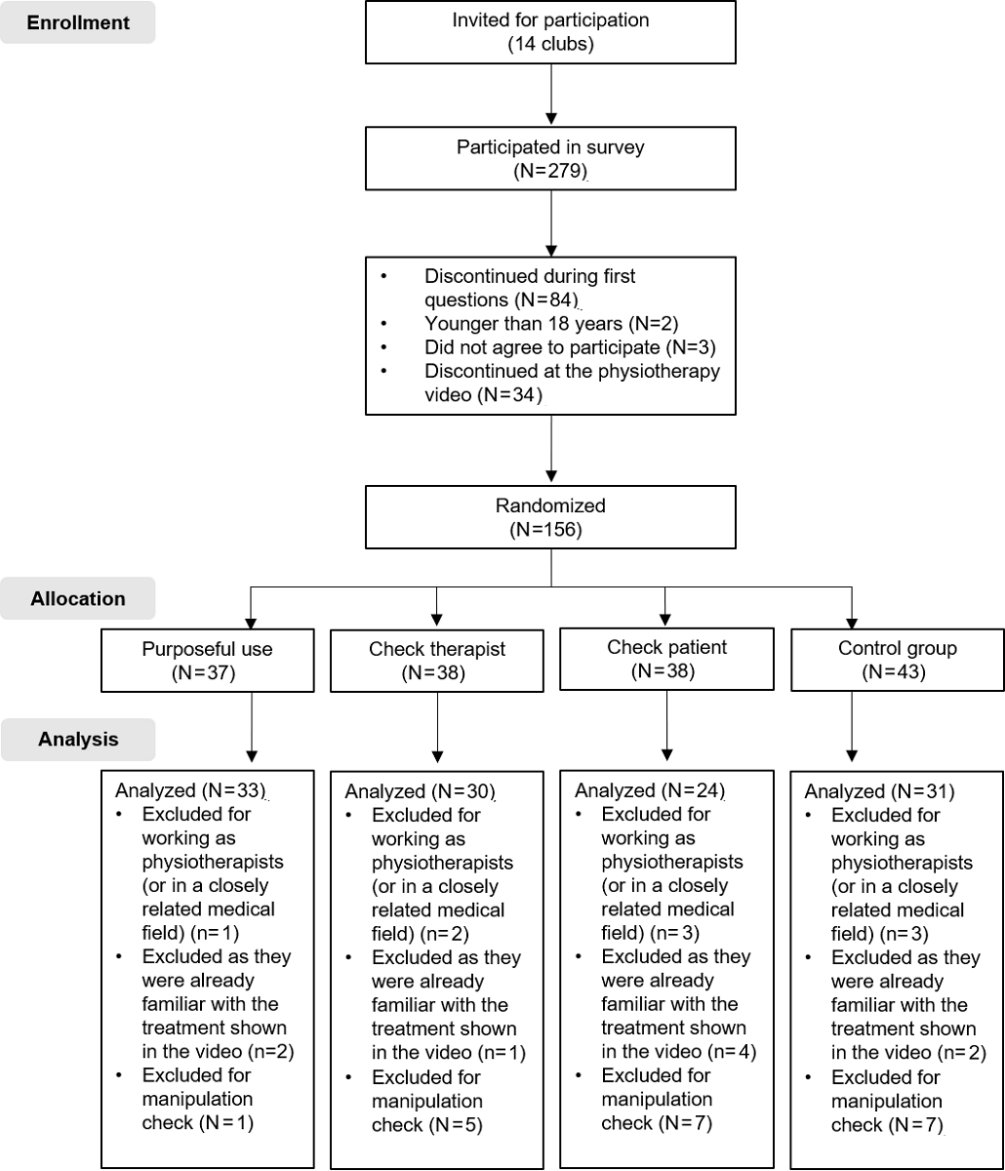
Flow diagram for study participants.

A power analysis for analysis of variance (ANOVA) with α=.05, an intended power of 95%, and a large effect size of 0.40 revealed a required sample size of 112. Members of various music and sports clubs in Germany (Federal State of Baden-Wuerttemberg) were invited via the clubs’ internal email distribution lists to participate from June to July 2019 in an online study. This was a relevant sample as both athletes and musicians often need physiotherapy [62]. The invitation email provided basic information about the study. This comprised the inclusion criteria (good German language skills and a minimum age of 18 years) and the formal requirements to participate in the study (the required technical device, ie, a computer, laptop, or smartphone with speakers or headphones, and the expected time required). A total of 279 people from 14 clubs took part in the survey. After applying the predefined exclusion criteria (Figure 2), we included 118 participants in the analysis. The age of the participants was between 18 and 74 years (mean 39.83, SD 15.15 years). Of the 118 participants, 63 stated they were female and 55 stated they were male. Additionally, 75 participants were employed or self-employed, 19 were students, three were in vocational training, eight were retirees, and 14 indicated other occupations, such as public officials. Seven participants did not answer the occupation question (multiple answers were possible). Most participants (n=107, 86.4%) indicated that they use their smartphones within the first hour after getting up, and more than half of the participants (n=61, 51.7%) indicated that they use their smartphones between 1 and 4 hours a day.

### Procedure

Initially, the participants provided information about their age, gender, current occupation, and daily smartphone usage. Thereafter, they were asked to imagine that they were undergoing physiotherapy for back pain. After that, the participants were randomly assigned to one of the four experimental conditions equally and they watched the appropriate video. Randomization was carried out by using Qualtrics software (Qualtrics). All participants were blinded to group allocation.

After watching the video, we conducted a manipulation check to ensure that the participants recognized the experimental treatment. They then indicated their perception of the professional competence, social competence, and empathetic behavior of the physiotherapist. They also rated the physiotherapist’s and patient’s attentiveness and evaluated the usage of smartphones in physiotherapy in general. Finally, the participants were debriefed. They were given the investigators’ contact information, and a link was provided to enable them to take part in a raffle for Amazon vouchers (two vouchers of €50 (USD 60) each and five vouchers of €20 (USD 23) each). Participation in the study took about 20 to 30 minutes.

### Measures

The online study was created using the Qualtrics software. The questionnaire of Willson and McNamara was used to measure *professional competence* and *social competence* [63]. This questionnaire compares eight pairs of adjectives to measure professional competence and nine pairs of adjectives to measure social competence on 9-point scales. These items are shown in Textbox 1. The reliability of the scales was determined by calculating the Cronbach alpha coefficient. There were good internal consistencies for the professional competence (α=.894) and social competence scales (α=.897).

Measurement of perceived professional and social competence. ^*^Reverse-coded items

**Professional competence**

Unprofessional (1) to professional (9)

Experienced (1) to inexperienced (9)^*^

Not thorough (1) to thorough (9)

Careful (1) to careless (9)^*^

Incompetent (1) to competent (9)

Trained (1) to untrained (9)^*^

Not appealing (1) to appealing (9)

Confident (1) to unconfident (9)^*^

**Social competence**

Friendly (1) to unfriendly (9)^*^

Impolite (1) to polite (9)

Attentive (1) to not attentive (9)^*^

Unkind (1) to kind (9)

Pleasant (1) to unpleasant (9)^*^

Not nice (1) to nice (9)

Caring (1) to not caring (9)^*^

Insensitive (1) to sensitive (9)

Sympathetic (1) to unsympathetic (9)^*^

*Empathetic behavior* of the physiotherapist was captured with a modified and shortened version of the CARE scale [64]. We modified the scale to fit the physiotherapeutic situation (rating the physiotherapist, not a physician). We used five items that participants rated on a 5-point Likert scale. Internal consistency for the scale was good (α=.867). The items are presented in Textbox 2.

**Measurement of empathetic behavior.**

1: The physiotherapist behaved in such a way that the patient felt comfortable around her.

2: The physiotherapist was caring and showed compassion to the patient.

3: The physical therapist really listened to the patient.

4: The physiotherapist encouraged the patient.

5: The physiotherapist explained everything to the patient in an understandable way.

We measured *attentiveness* with six self-created items. Three questions focused on the perceived attentiveness of the patient, and another three focused on the perceived attentiveness of the therapist (Textbox 3). The data were recorded using a 5-point Likert scale. There was good internal consistency for both scales (attentiveness of the patient: α=.830; attentiveness of the physiotherapist: α=.800).

Measurement of the perceived attentiveness of the physiotherapist and the patient. ^*^Reverse-coded items.

**Attentiveness of the physiotherapist**

-The therapist seemed to be distracted during the therapy.^*^

-The therapist’s attention was completely focused on the therapy.

-The therapist showed great interest in the progress of the therapy.

**Attentiveness of the patient**

-The patient seemed to be distracted during the therapy.^*^

-The patient’s attention was completely focused on the therapy.

-The patient showed great interest in the progress of the therapy.

We also measured how participants evaluated the use of smartphones in physiotherapy in general (Textbox 4). The data were collected using a 5-point Likert scale. The internal consistency was good (α=.895).

Evaluation of the usage of smartphones in physiotherapy. ^*^Reverse-coded items.

1: A smartphone is mainly distracting in physiotherapy.^*^

2: A smartphone interferes during physiotherapy.^*^

3: A smartphone stands in the way of good physiotherapy.^*^

4: It is very useful to use a smartphone in physiotherapy.

5: The use of a smartphone in physiotherapy is very supportive.

6: Using a smartphone in physiotherapy entails advantages.

### Analysis

Data analysis was performed using IBM SPSS 25 statistics (IBM Corp) for Windows. To test our hypotheses and answer the open research questions, we performed contrast analysis. Contrast analysis allows for testing specific hypothesized patterns of mean differences by defining lambda coefficients while increasing statistical power and avoiding issues of multiple testing, which would arise with pairwise comparisons using *t* tests [65]. In addition, contrast analysis produces distinct test statistics for situations in which the group variances are equal or unequal. To decide for the correct test statistic, we used the Levene test for homogeneity of variance. To test the comparability of the conditions, ANOVA and chi-square tests were performed.

We provide mean, SD, and R as an indicator of effect size for significant results. According to a previous report [66], we interpreted *R*=0.10 as a small effect, *R*=0.20 as a typical effect, and *R*=0.30 as a relatively large effect. The significance level for all analyses was set to α=.05.

## Results

### Comparability of the Conditions

There were no significant differences among the four experimental conditions regarding participants’ sex (*χ*^2^_3_=2.02, *P*=.57), age (*F*_3,114_=0.68, *P*=.56), and smartphone usage (average time of smartphone usage: *P*=.73; time interval between getting up in the morning and the first use of the smartphone: *P*=.13).

### Hypothesis Testing

**Figure 3:**
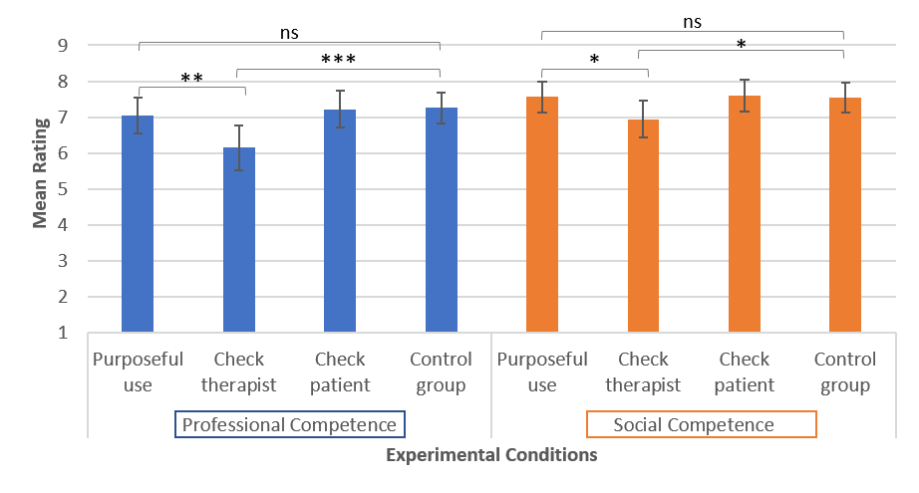
Means and CIs for the outcome variables professional competence and social competence in the four experimental conditions. ns: nonsignificant. *P<.05, **P<.01, ***P<.001.

**Figure 4:**
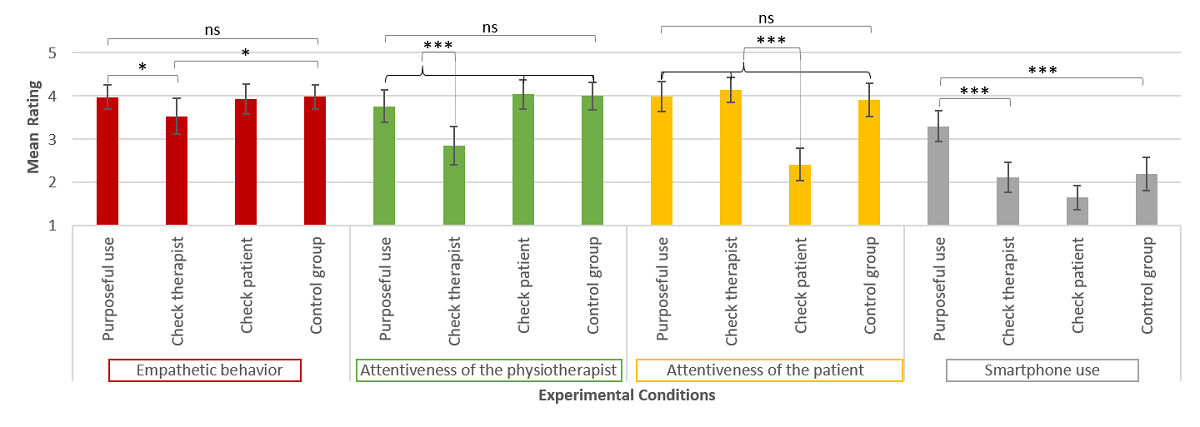
Means and CIs for the outcome variables empathetic behavior, attentiveness of the physiotherapist, attentiveness of the patient, and smartphone use in the four experimental conditions. ns: nonsignificant. *P<.05, ***P<.001.

All of the outcome variables (professional competence: *P*=.14; social competence: *P*=.13; attentiveness of the physiotherapist: *P*=.08; attentiveness of the patient: *P*=.39; smartphone use: *P*=.11), except empathetic behavior (*P*=.01), met the criteria of variance homogeneity. The scores for all of the experimental conditions are shown in Figure 3 and Figure 4.

In hypothesis 1, we assumed that the professional competence of the physiotherapist would be perceived as more pronounced if the physiotherapist used the smartphone in a purposeful way than if the physiotherapist looked at it from time to time with no therapeutic purpose. A contrast analysis supported this assumption (t_112_=2.53, *P*=.007, *R*=0.23). We found the same pattern of results for the perception of social competence (hypothesis 2; t_112_=1.99, *P*=.03, *R*=0.19) and empathy (hypothesis 3; t_49.17_=1.82, *P*=.04, *R*=0.25).

Hypothesis 4a was supported by the data as well. When the physiotherapist used the smartphone in a recreational way, the physiotherapist was perceived as being less attentive than in all of the other conditions (t_105_=5.15, *P*<.001, *R*=0.45). The same applied to hypothesis 4b that was concerned with the attentiveness of the patient. When the patient used the smartphone in a recreational way, the patient was also perceived as being less attentive than in all of the other conditions (t_105_=7.63, *P*<.001, *R*=0.60).

In line with hypothesis 5, we found that the way the smartphone was used had an impact on participants’ evaluation of smartphone use in physiotherapy in general. If the physiotherapist used the smartphone in a purposeful way, smartphone use in physiotherapy was rated more positively compared with the conditions in which the physiotherapist or the patient looked at the smartphone from time to time with no therapeutic purpose (t_102_=7.01, *P*<.001, *R*=0.57).

### Open Research Questions

As further contrast analyses showed, differences in perceived professional competence, perceived social competence, and perceived empathetic behavior occurred owing to the fact that the checking behavior of the physiotherapist led to a lower rating than no smartphone use (professional competence: t_112_=3.12, *P*=.001, *R*=0.28; social competence: t_112_=1.93, *P*=.03, *R*=0.18; empathy: t_49.54_=1.86, *P*=.04, *R*=0.26).

Purposeful use of the smartphone did not lead to a higher rating of perceived professional competence (*P*=.27), perceived social competence (*P*=.48), or perceived empathetic behavior (*P*=.47) in comparison with no smartphone use. The positive evaluation of smartphone use in physiotherapy occurred owing to the fact that purposeful use of the smartphone led to a more positive rating in comparison with no smartphone use (t_102_=4.68, *P*<.001, *R*=0.42) and not because use of the smartphone with no therapeutic purpose led to a less positive rating than no smartphone use (*P*=.38).

## Discussion

### Overview

This study examined to what extent smartphone use in a physiotherapeutic treatment session impacted the perceived competence and empathy of the physiotherapist, the perceived attentiveness of the physiotherapist and the patient, and the overall evaluation of the use of smartphones in physiotherapy. To our knowledge, this is the first empirical investigation into the effects of different kinds of smartphone usage in physiotherapeutic treatment situations. By examining the difference between purposeful use of smartphones and the often unconsciously occurring checking (or phubbing) behavior, we aimed to contribute to the highly socially relevant discussion about new interaction phenomena induced through the omnipresence of smartphones.

### General Findings

As expected, our results indicated that purposeful use of a smartphone by a physiotherapist was assessed differently from recreational smartphone use regarding the rating of the physiotherapist’s professional and social competence and empathetic behavior. Interestingly, this was the case because the checking behavior of the physiotherapist led to a lower rating than no smartphone use and not because of the superiority of purposeful smartphone use. It seems that the smartphone used as a treatment tool was accepted, but it did not lead to any more positive perceptions of the therapist. It is therefore particularly important to use a smartphone in therapy only in a targeted manner; otherwise, it can have a negative impact on the physiotherapist-patient relationship, which is important for treatment success [46,54]. Regarding the perceived attentiveness of the physiotherapist and patient, the smartphone checking behavior again had a negative influence. Nevertheless, it was shown that a positive evaluation of smartphone usage in physiotherapy in general occurred owing to the fact that purposeful use of the smartphone led to a more positive rating in comparison with no smartphone use or checking behavior. This clearly showed that the participants saw the potential of using smartphones to improve treatment quality, but they also felt that inconsiderate use entailed hindrance of the social interaction in physiotherapy.

Overall, the results of our study are in line with other research findings demonstrating that smartphone use in social situations can have negative effects on social interactions [24,51,67] and perceived interpersonal attention [68]. However, the study also showed a possible remedy for the negative consequences of smartphone use, that is, utilizing the smartphone with a clear purpose can be experienced and appreciated by communication partners and patients.

### Limitations

Our research has some limitations worth noting. First, the experiment conducted here only relied on video material of a physiotherapeutic treatment situation. While videos can be a great format for illustrating processes, they are only an imitation of real treatment situations. Owing to the exact scripting of the video, we had the advantage of creating standardized experimental material, but the disadvantage of a reduced level of realism. In addition, the participants acted as observers and not as patients or therapists. We cannot know conclusively whether actual interaction partners in situations like the one in the video would perceive the situation in the same way. Moreover, this study cannot make a statement about whether a certain level of smartphone use is necessary to achieve certain effects, whether there must be a minimum threshold of smartphone use, or whether more smartphone use leads to stronger effects. The findings of this study only allow conclusions to be drawn about whether a certain type of use in general caused the effects investigated. Another limitation was the relatively small sample size. As the power analysis showed, we could only determine rather large effects. Large effects are also more relevant to clinical practice. Owing to the sample size, we cannot draw any conclusions on specific participant characteristics, such as gender and age. This should be addressed in further studies. It would also be interesting to examine different characteristics (eg, gender, age, and digital competence) of the therapist in further studies. Finally, we considered only one physiotherapeutic treatment situation. Generalization to other treatments or medical conditions is therefore not possible. We advise researchers to examine the effects of different kinds of smartphone uses in real clinical settings in the future.

### Conclusions

This research showed that the frequently reported negative effects of smartphone use in social (face-to-face) interactions were not perceived as such by our participants if the smartphone was used in an obviously purposeful way. In this kind of usage, participants even see the positive potential of smartphone use in physiotherapy. In summary, we recommend that practitioners use smartphones in a medical treatment situation only as actively integrated supportive tools for the purpose of treatment. Otherwise, a smartphone should be used with great awareness, and checking behavior should be avoided as often as possible.

## Appendix

Multimedia Appendix 1 CONSORT checklist.

## Abbreviations

ANOVA: analysis of variance
